# Hippocampal glutamate and hippocampus subfield volumes in antipsychotic-naive first episode psychosis subjects and relationships to duration of untreated psychosis

**DOI:** 10.1038/s41398-020-0812-z

**Published:** 2020-05-12

**Authors:** Frédéric Briend, Eric A. Nelson, Omar Maximo, William P. Armstrong, Nina V. Kraguljac, Adrienne C. Lahti

**Affiliations:** 1 Department of Psychiatry and Behavioral Neurobiology, University of Alabama at Birmingham, Birmingham, AL USA; 2 Department of Psychology, University of Alabama at Birmingham, Birmingham, AL USA

**Keywords:** Schizophrenia, Molecular neuroscience

## Abstract

Evidence points toward a relationship between longer duration of untreated psychosis (DUP) and worse long-term outcomes in patients with first episode psychosis (FEP), but the underlying neurobiology remains poorly understood. Proton magnetic resonance spectroscopy studies have reported altered hippocampus glutamatergic neurotransmission, and structural MRI as reported hippocampal atrophy that may be associated with memory impairment in schizophrenia. Here, we quantify left hippocampus glutamate (Glx) and left hippocampus subfield volumes in 54 antipsychotic-naive FEP and 41 healthy controls (HC), matched on age, sex, and parental occupation. While there were no significant group difference in Glx levels, hippocampal Glx levels were significantly higher in those who underwent a long DUP (>12 months) compared to those with a short DUP, and compared to HC. Compared to HC, FEP had significantly reduced whole hippocampus volume, as well as of CA1, CA4, granule cell layer, subiculum, and presubiculum subfields. Smaller whole hippocampal volume, as well as CA1, molecular layer, subiculum, presubiculum, and hippocampal tail volumes were significantly associated with longer DUP. However, we found no significant association between hippocampal Glx levels and hippocampal volume or subfields, suggesting that these alterations are not related, or their relationship does not follow a linear pattern. However, our results strongly suggest that one or several pathophysiological processes underlie the DUP. Importantly, our data highlight the critical need for reducing the DUP and for early pharmacological intervention with the hope to prevent structural deficits and, hopefully, improve clinical outcomes.

## Introduction

Meta-analyses have consistently identified an association between longer duration of untreated psychosis (DUP), the duration between the onset of positive symptoms and treatment, and worse clinical outcome^1^. Longer DUP is associated with worse negative symptoms at first treatment contact, and with poorer symptomatic and functional recovery from the first psychotic episode^1–4^. The relationship between DUP and outcome is found across various lengths of follow-up periods, suggesting DUP influences the long-term course of the illness^4^. In addition, there is evidence that the association of DUP with outcome is more pronounced for longer DUP (>12 months) compared to shorter DUP (ref. ^4^). A deleterious effect of psychosis has been hypothesized; later it was proposed that hypofunction of *N*-methyl-D-aspartate receptors on gamma-aminobutyric acid (GABA) inhibitory neurons results in a hyperactivation of glutamate neurons, leading to excess glutamate release, and, if the disruption is sustained, neuronal insult^5,6^.

The hippocampus plays a key role in memory processes, especially in long-term episodic memory^7^, which has been shown to be impaired in schizophrenia (SZ)^8^. At the cytoarchitectonical level, hippocampal subdivisions include the subiculum complex, cornu ammonis (CA) 1–4, and the dentate gyrus (DG)^9^. At the neuronal level, the pyramidal layers of the hippocampus are tightly packed with glutamatergic neurons that have a low firing threshold, ensuring a high level of neuroplasticity in the region^10^. The glutamatergic pyramidal neurons account for ~90% of hippocampal neurons, a much higher percentage than in other parts of the cortex^11^. The remaining 10% of hippocampal neurons consist of inhibitory GABAergic interneurons tasked with regulation of the easily excited glutamatergic neurons^10,12^. Postmortem studies in SZ have indicated normal hippocampal pyramidal neuronal density and number^13,14^, but reduced number of GABAergic interneurons^15^. In addition, the GABA-synthesizing enzymes glutamic acid decarboxylase (GAD)65 and GAD67 are decreased in the hippocampus in SZ (ref. ^16^).

Converging lines of evidence from memory assessments^8^, molecular studies^17,18^ to multimodal brain imaging^19–21^ points to hippocampal alterations in SZ. In addition, hippocampal dysfunction is at the root of some prominent neurobiological models of SZ (refs. ^10,22,23^). Hippocampal alterations have been identified in family members^24^, and those at risk to develop psychosis^25^. More generally, hippocampal dysfunction has also been described in psychotic disorders^2,21,25–30^, suggesting this dysfunction plays a key role in etiology of the psychotic disorders.

Consistent with a deleterious effect of psychosis on brain structure, a recent study reported that, in FEP, longer DUP was associated with accelerated hippocampal atrophy over the initial 8 weeks of antipsychotic treatment, suggesting a persistent effect of DUP on brain structure^31^. In addition, we previously reported elevated hippocampal glutamate + glutamine (Glx) levels in a group of unmedicated patients with SZ (refs. ^32–34^), and an association between decreased hippocampal volume and increased Glx (ref. ^32^). Furthermore, clinical outcomes in individuals at clinical high risk for psychosis may be associated with an increase in baseline hippocampus Glx levels^35^. Taken together, these studies suggest that hippocampal dysfunction plays a critical role in the onset of psychosis; however, the biologic basis for this dysfunction and its relationship to the DUP have not been identified^36^.

In this study, we obtained measurements of hippocampal Glx and hippocampal volume segmentation in a group of antipsychotic-naive FEP and matched healthy controls (HC). We systematically assessed the DUP based on information provided by the patient and their caregivers during screening and, at any time during the 32-week follow-up. Based on the existing literature, we hypothesized that we would observe higher Glx levels in FEP compared to controls. We further hypothesized that, in FEP, longer DUP would be associated with elevated Glx levels, and with smaller whole hippocampal and subfield volumes. Finally, we hypothesized that in FEP higher Glx levels would be associated with smaller hippocampal volumes, and mediate the association between DUP and smaller whole hippocampus and subfield volumes. Finally, we also conducted exploratory analyses to investigate associations with clinical and behavioral memory measures.

## Material and methods

### Participants

Sixty-six antipsychotic-naive FEP subjects were recruited from the emergency department, inpatient units, and outpatient psychiatry clinics at the University of Alabama at Birmingham (UAB). Seven patients dropped out for the following reasons: they withdrew consent before scan (*n* = 2), they did not tolerate the scan environment (*n* = 3) or for unknown reason (*n* = 2).

Diagnoses were established according to DSM-V criteria by review of medical records and consensus of two board-certified psychiatrists (A.C.L. and N.V.K.). Forty-one HC, matched on age, gender, and parental socioeconomic status were recruited by advertisements. Exclusion criteria were major neurological or medical conditions, history of significant head trauma, substance use disorders (excluding nicotine and cannabis) within 1 month of imaging, >5 days of lifetime antipsychotic exposure, pregnancy or breastfeeding, and MRI contraindications. HC with a personal or family history in a first-degree relative of a psychiatric disorder were excluded. The UAB Institutional Review Board gave approval for this study, and written informed consent was obtained prior to enrollment and after subjects were deemed to have capacity to provide consent^37^.

### Duration of untreated psychosis

DUP was defined as the duration between the first onset of discernable positive symptoms to the time of initial treatment contact (the time at which the first antipsychotic prescription was written, which also coincided with enrollment in the study) and is reported in months. Two experienced psychiatrists systematically assessed the DUP based on information provided by the patient and their caregivers during screening, and at any time during the follow-up period. Moreover, we dichotomized DUP into short (<12 months) and long (>12 months) based on a number of studies that used this cutoff to define long and short DUP (refs. ^4,38–41^), including those predicting treatment outcomes in early psychosis^42^ and poorer outcomes after the first hospitalization^43^.

### Clinical and behavioral scales

The brief psychiatric rating scale (BPRS) was used for assessments of symptom severity^44^. The repeatable battery for the assessment of neuropsychological status (RBANS) was used to characterize memory cognitive function of our subject, via their total, immediate, and delayed memory subscales^45^.

### Image acquisition of the proton magnetic resonance spectroscopy

**Fig. 1:**
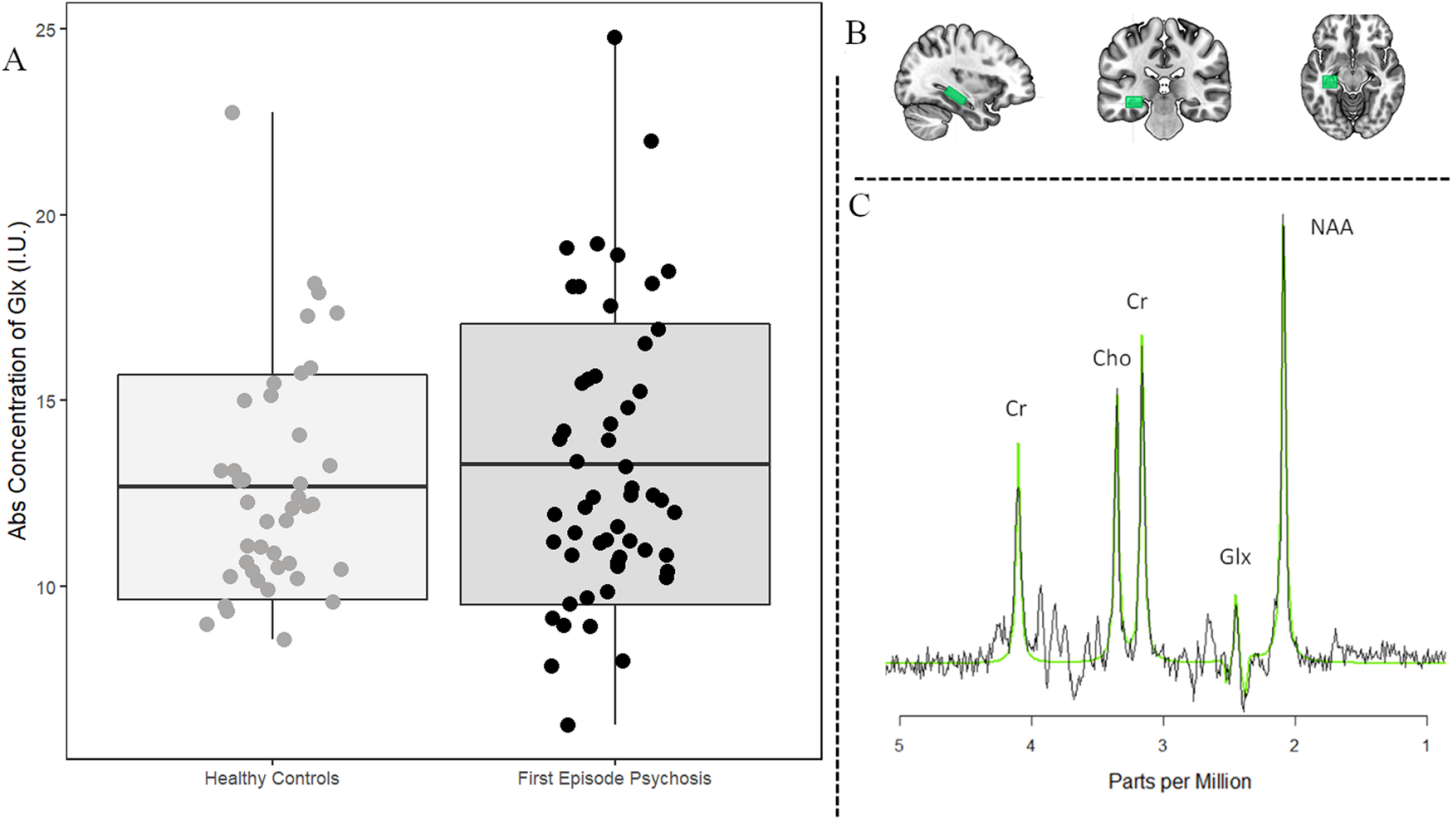
Proton magnetic resonance spectroscopy of the left hippocampus. **a** Boxplot of glutamate level in antipsychotic-naive first episode psychosis patients (FEP) compared with healthy controls (HC; *P* = 0.39). Each point corresponds to one participant (mean ± standard deviation). HC: *n* = 41, FEP: *n* = 54. **b** Single voxel location in the left hippocampus. **c** Spectrum from left hippocampus volume of interest; the black line is a collected spectrum and the green line is a model fit obtained by the AMARES algorithm in jMRUI. Cr indicates creatine, Cho choline, Glx glutamate and glutamine, and NAA *N*-acetyl aspartate.

Data were collected from a voxel in the left hippocampus, such that the amount of gray matter was maximized while avoiding major vessels (27 × 15 × 10 mm^3^; Fig. 1). Following automatic and manual shimming to optimize field homogeneity across the voxel, chemical shift selective pulses were used to suppress the water signal. Then, spectra were obtained using a point-resolved spectroscopy sequence (TR/TE = 2000/80 ms, flip angle = 90°, vector size 1024, 192 averages; the echo time was chosen in order to resolve and separate the C4 resonance of Glx from other *j*-coupling metabolites^46,47^). Moreover, eight averages of unsuppressed water scans with the same acquisition parameters were acquired for quantify metabolite according to the water peak.

### Proton magnetic resonance spectroscopy data processing

All spectra were analyzed in jMRUI version 6.0 using the AMARES algorithm^48^. Prior knowledge derived from in vitro and in vivo spectra was included in the model. A phantom solution of 20 mM glutamate in buffer (30 mM sodium hydrogen carbonate and 30 mM sodium carbonate; pH, 7.1) was imaged using the same acquisition parameters from the in vivo study. The model consisted of peaks for *N*-acetyl aspartate, choline, creatine (Cr + Cr2), and Glx was modeled as a triplet (large peak with two small outer wings), as previously described^32^. After removing the residual water peak using the Hankel-Lanczos singular values decomposition filter, the amplitude of the center Glx peak was estimated, and Glx levels were calculated relative to the unsuppressed voxel water and expressed in institutional units^49^. Metabolite levels were corrected for partial volume effects according to Gasparovic and colleagues^50,51^; the fraction of cerebrospinal fluid, gray, and white matter were calculated by segmentation of the T1-weighted images in SPM 8 (see in Supplementary Data).

Exclusion criteria for Glx were failure of the fitting algorithm, signal to noise ratio < 3, full-width at half-maximum > 0.1 ppm (ref. ^52^), and Cramer–Rao lower bounds > 20%. Five FEP subjects were excluded from the analyses based on those criteria.

### Hippocampus subregions segmentation

Each participant’s high-resolution T1- and T2-weighted images were preprocessed using the FreeSurfer 6.0 (ref. ^53^). All structural images underwent skull stripping via FSL (ref. ^54^) before submission to the recon-all preprocessing pipeline^53^. Following this step, we used Freesurfers’ hippocampus subfield segmentation module to calculate each participants left and right subregions volumes^55^. Freesurfer 6.0 has a significantly improved segmentation algorithm that uses Bayesian inference combined with a manually delineated hippocampal atlas^55^. The estimated total intracranial volume (ICV) was also obtained via FreeSurfer 6.0 (ref. ^56^; Table 1).

**Table 1:** Demographic and clinical information of the sample.

	HC	FEP all	FEP subgroup *n* = 54		*t*-Test (HC vs FEP)	
			Long DUP	Short DUP		*t*-Test (long vs short)
	*n* = 41	*n* = 54	*n* = 17	*n* = 37		
Age (years)	24.75 (6.35)	23.88 (6.16)	24.58 (6.63)	23.56 (6.00)	*P* = 0.50; *d* = 0.14	*P* = 0.57; *d* = 0.16
Gender (% males)	60.97	64.81	58.82	67.56	Chi^2^: 0.86	Chi^2^: 0.07
Socioeconomic status (SES)	4.21 (4.20)	6.03 (5.05)	5.62 (5.28)	6.22 (5.01)	*P* = 0.09; *d* = −0.38	*P* = 0.70; *d* = −0.12
Smoking status (pack-day)	0.015 (0.07)	0.21 (0.34)	0.18 (0.34)	0.22 (0.34)	*P < 0.01; *d* = −0.74	*P* = 0.66; *d* = −0.13
Duration of untreated psychosis (weeks)	—	19.93 (36.60)	55.76 (49.18)	3.47 (3.58)	—	*P < 0.01; *d* = 1.90
Intracranial volume (mm^3^)	10,000,907 (118056.2)	985,471.4 (126406.7)	1,002,273 (133221.7)	987,526.5 (117958.4)	*P* = 0.54; *d* = 0.12	*P* = 0.62; *d* = 0.12
Brief psychiatric rating scale (BPRS)						
Positive	—	11.50 (3.31)	10.41 (3.26)	12 (3.25)	—	*P* = 0.11; *d* = −0.48
Negative	—	5.92 (3.22)	5.11 (2.49)	6.29 (3.47)	—	*P* = 0.16; *d* = −0.36
Total	—	49.90 (11.03)	46.88 (10.24)	51.29 (11.23)	—	*P* = 0.16; *d* = −0.40
RBANS						
Total	94.06 (10.29)	75.69 (15.93)	81.07 (18.83)	73.24 (14.05)	*P < 0.01; *d* = 1.31	*P* = 0.16; *d* = 0.50
Immediate memory	102.87 (15.57)	83.14 (19.03)	84.53 (19.83)	82.51 (18.94)	*P < 0.01; *d* = 1.11	*P* = 0.74; *d* = 0.10
Delayed memory	92.58 (8.72)	77.41 (16.34)	83.40 (16.89)	74.70 (15.60)	*P < 0.01; *d* = 1.09	*P* = 0.10; *d* = 0.54

*HC* healthy controls, *FEP* first episode psychosis’s patients, *DUP* duration of untreated psychosis (long > 12 months > short), *SES* parental socioeconomic ranks determined from diagnostic interview for genetic studies (1–18 scale), *BPRS* brief psychiatric rating scale, *RBANS* repeatable battery for the assessment of neuropsychological status, *d* Cohen’s *d*.

We assessed the overall hippocampal volume, and considered eight subregions across allocortical regions of the hippocampal formation^29^: the hippocampal tail (comprised of portions of CA and DG), CA1, CA3 (CA2 and CA3 are combined in the Freesurfer atlas), CA4, molecular layer (ML) of the subiculum sub volumes and the DG, the granule cell layer of DG (GC-ML-DG: a combination of the granular cell layer, the ML, and the DG), and finally subiculum subfields (subiculum and presubiculum). These regions have been found to be altered in SZ (refs. ^21,27,30^). Smaller area, as the fimbria, the hippocampal fissure and the parasubiculum were not included in this study due to reliability concerns in their segmentations^21,57^.

Data quality was assessed using the Qoala-T-tool^58^ that uses a supervised-learning model to assess accuracy of manual quality control of automated segmentation. Each participant’s subfield segmentation was assessed by visual inspection (E.N. and O.M.), no hippocampal segmentation failures were reported.

### Statistical analysis

Statistical analyses were performed in R (http://cran.r-project.org).

To compare Glx and hippocampus volumes between groups, we used a two-sample *t*-test (FEP vs HC) and a one-way ANCOVA to compared HC, long and short DUP, controlling for age, sex, and smoking status (pack-day). To assess the relationship between Glx, hippocampus volume measures, and clinical variables, we used partial correlations with age, sex, and smoking status included as covariates. For any analyses, including hippocampus volume, we included ICV as an additional covariate.

To complement analyses, we also calculated Cohen’s *d* effect sizes. All analyses were controlled for multiple comparisons using the false detection rate (FDR) correction method by Benjamini and Hochberg^59^.

## Results

FEP and HC were well matched in terms of age, gender, and socioeconomic status. However, they differ in smoking status (Table 1, *P* < 0.01).

### Glx levels in HC, FEP, and relationship with DUP

There was no significant group difference in Glx levels in the left hippocampus (Fig. 1c, Cohen’s *d* = −0.17; *t*(94) = −0.86; *P* = 0.39).

**Fig. 2:**
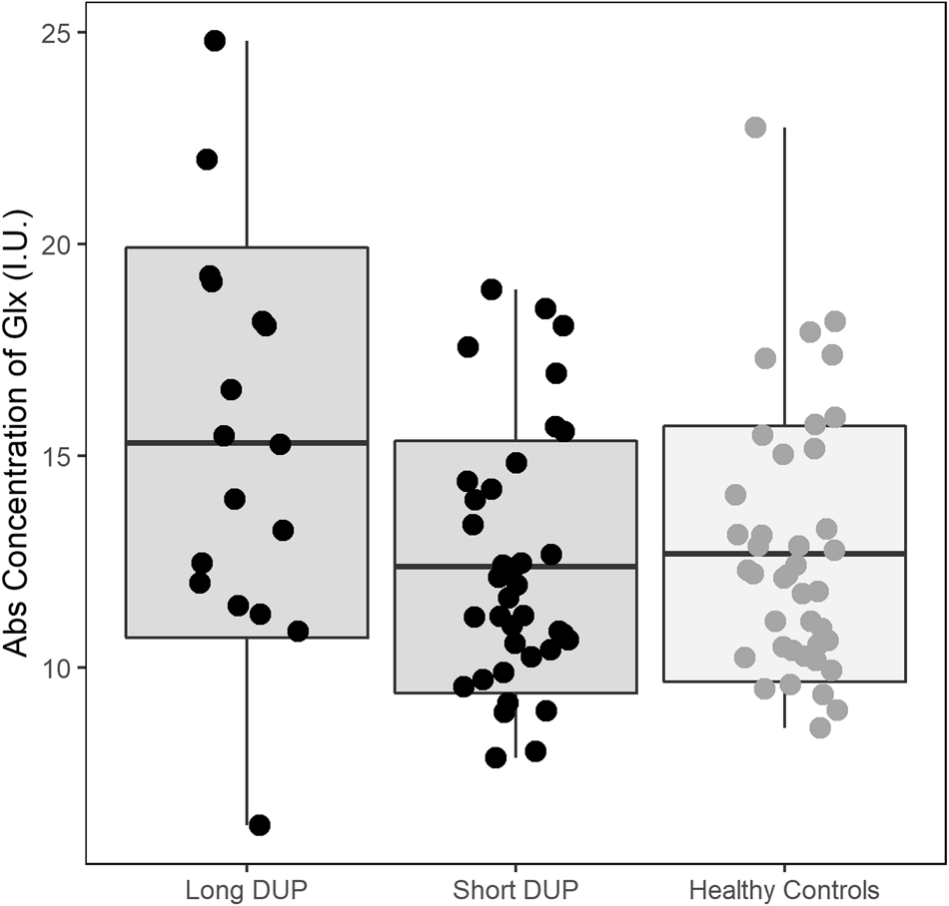
Left hippocampus glutamate (Glx) level in the divided group of antipsychotic-naive FEP patient compared to HC. Those with a longer duration of untreated psychosis (DUP; >12 months) showed an increase of Glx concentration compared to those with a short DUP (*P* < 0.02) and to HC (*P* = 0.03). Each point corresponds to one participant (mean ± standard deviation). Long DUP: *n* = 17, short DUP: *n* = 37, and HC: *n* = 41.

Although Glx levels were not significantly correlated with DUP in FEP (*r* = 0.22, *P* = 0.11), when patients were dichotomized into those with long and short DUP based on a cutoff point of 12 months, the ANOVA revealed a significant group effect (*F*(2,89) = 4.92; *P* < 0.01). Those with long DUP (*n* = 17) had significantly higher Glx levels than those with shorter DUP (*n* = 37; Fig. 2, Cohen’s *d* = −0.82; *t*(89) = 3.14; *P* < 0.01) and than HC (Cohen’s *d* = −0.74; *t*(89) = 2.67; *P* = 0.03).

### Hippocampus volume in HC, FEP, and relationship with DUP

Left whole hippocampus volume was significantly reduced in FEP compared to HC (Cohen’s *d* = 0.59; *t*(85.7) = 2.87; *P* < 0.01). We also found lower volumes in the following hippocampus subfields: subiculum (Cohen’s *d* = 0.53; *t*(85.7) = 2.53; *P* = 0.03), presubiculum (Cohen’s *d* = 0.71; *t*(85.7) = 3.41; *P* < 0.01), CA1 (Cohen’s *d* = 0.62; *t*(85.7) = 3.04; *P* = 0.01), GC-ML-DG (Cohen’s *d* = 0.48; *t*(85.7) = 2.35; *P* = 0.04), and CA4 (Cohen’s *d* = 0.45; *t*(85.7) = 2.21; *P* = 0.04; *P* < 0.05 FDR corrected; Table 2). Similar group differences were obtained for the right hippocampus (Supplementary Table 1). Although left and right whole hippocampus volumes, and all the subregions are reduced in long DUP compared to short DUP, these did not survive multiple comparisons (Table 2, Supplementary Table 1).

**Table 2:** Statistical comparisons of left hippocampal subfield volumes (mm^3^) in FEP and HC.

	HC (*n* = 41)	FEP (*n* = 54)	*t*(85.7)	*P* at FDR 0.05	Cohen’s *d*
Left whole hippocampus	3483.54 (366.9)	3271.4 (346.5)	2.87	<0.01*	0.59
Left hippocampal tail	581.15 (76.80)	553.36 (63.65)	1.89	0.06	0.4
Left subiculum	440.43 (56.17)	413.38 (47.35)	2.53	0.03*	0.53
Left cornu ammonis 1	647.20 (79.45)	596.62 (82.81)	3.04	0.01*	0.62
Left presubiculum	314.66 (39.06)	288.11 (36.14)	3.41	<0.01*	0.71
Left molecular layer	520.10 (961.86)	464.92 (56.83)	2.04	0.06	0.42
Left GC-ML-DG	307.43 (36.76)	289.79 (36.16)	2.35	0.04*	0.48
Left cornu ammonis 3	212.89 (29.70)	201.30 (28.91)	1.91	0.06	0.39
Left cornu ammonis 4	253.50 (29.55)	239.94 (30.04)	2.21	0.04*	0.45

	FEP subgroup				
	Long DUP (*n* = 17)	Short DUP (*n* = 37)	*t*(26.42)	*P* at FDR 0.05	Cohen’s *d*
Left whole hippocampus	3171.42 (388.6)	3317.41 (320.60)	1.45	0.18	−0.42
Left hippocampal tail	538.24 (78.03)	560.30 (55.70)	−1.05	0.46	−0.35
Left subiculum	395.56 (46.68)	421.13 (46.04)	−1.87	0.36	−0.55
Left cornu ammonis 1	574.93 (89.01)	606.58 (79.06)	−1.25	0.46	−0.38
Left presubiculum	275.98 (33.83)	293.68 (36.24)	−1.74	0.36	−0.51
Left molecular layer	480.73 (63.31)	501.42 (53.25)	−1.17	0.46	−0.37
Left GC-ML-DG	282.95 (43.38)	292.94 (32.50)	−0.87	0.46	−0.27
Left cornu ammonis 3	197.35 (31.15)	203.12 (28.08)	−0.65	0.52	−0.21
Left cornu ammonis 4	234.06 (35.81)	242.64 (27.11)	−0.88	0.46	−0.28

All statistical analyses were controlled for multiple comparisons by false detection rate correction method.

*HC* healthy controls, *FEP* first episode psychosis’s patients, *DUP* duration of untreated psychosis (long >12 months > short), *GC-ML-DG* granular cell of the dentate gyrus.

**Fig. 3:**
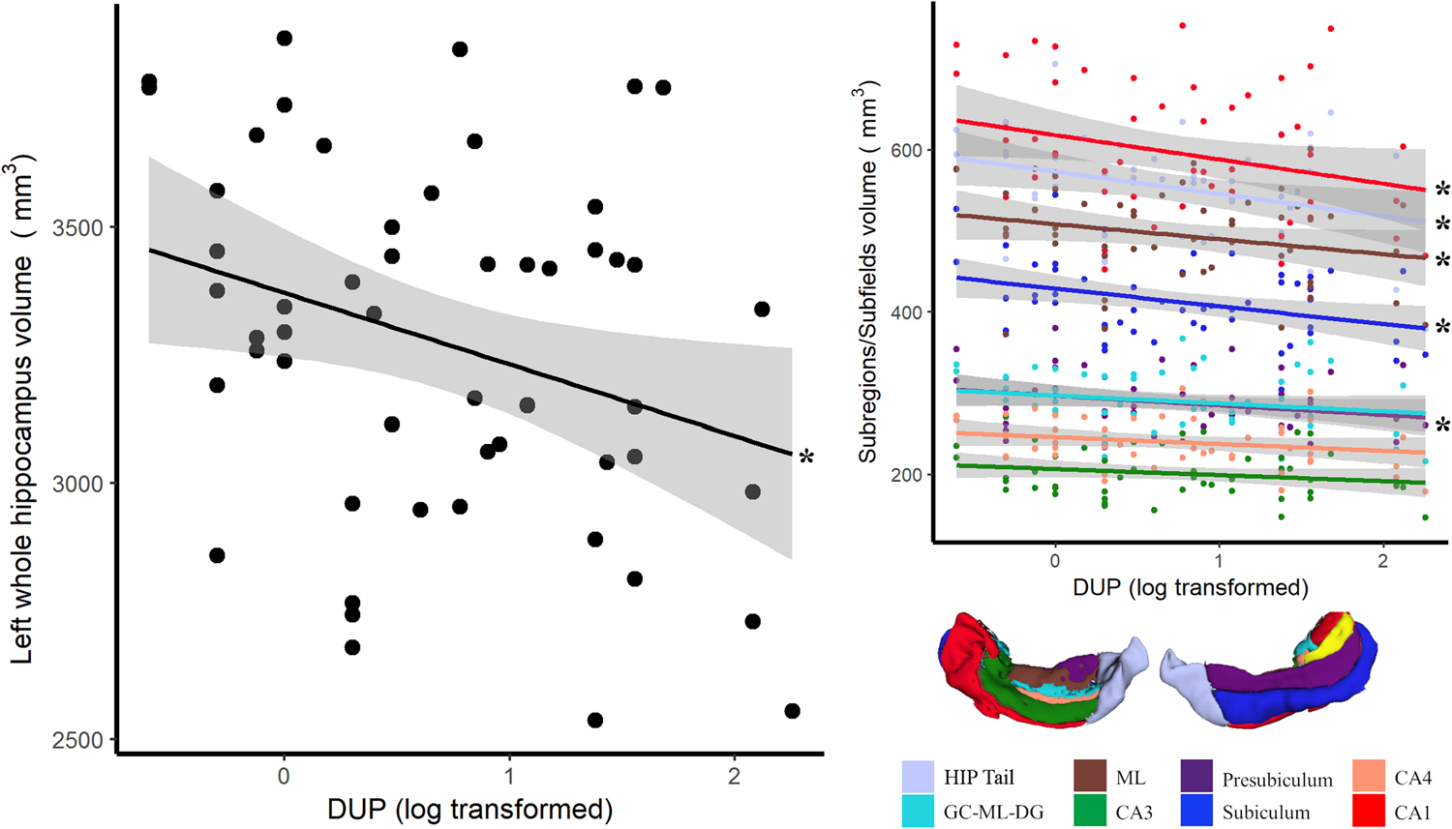
Association of left hippocampus volume and DUP in antipsychotic-naive FEP patients. Left: relationship between the whole left hippocampus volume and the DUP, controlling for age, gender, and total ICV (*r* = −0.37, *P* < 0.01). Right: association between the left hippocampus subregions volumes and DUP, controlling for age, gender, and total ICV. Medial view of the left hippocampus volumes (left: anterior to posterior, right: posterior to anterior). *P < 0.05. Each point corresponds to one participant. HIP hippocampus, CA cornu ammonis, GC-ML-DG granular cell of the dentate gyrus, ML molecular layer. FEP: *n* = 54.

In FEP subjects, longer DUP was significantly associated with smaller whole hippocampus volume (Fig. 3, Table 3, *r* = −0.37, *P* < 0.01). This relationship was also seen in the following subfields: hippocampus tail (*r* = −0.32, *P* = 0.04), CA1 (*r* = −0.31, *P* = 0.04), ML (*r* = −0.30, *P* = 0.04), subiculum (*r* = −0.41, *P* = 0.02), and presubiculum (*r* = −0.33, *P* = 0.04) volumes.

**Table 3:** Correlations between left hippocampus subfield volumes and DUP, Glx level.

	HC	FEP all	FEP subgroup		
			Long DUP	Short DUP	
	*n* = 41	*n* = 54	*n* = 17	*n* = 37	*t*-Test (long vs short)
*Hippocampus subfield volumes and DUP*					
Left whole hippocampus	—	*r* = −0.37, *P* < 0.01*	*r* = −0.05, *P* = 0.86	*r* = −0.38, *P* = 0.02*	*P* = 0.18; *d* = −0.42
Left hippocampal tail	—	*r* = −0.32, *P* = 0.04*	*r* = −0.26, *P* = 0.96	*r* = −0.37, *P* = 0.13	*P* = 0.46; *d* = −0.35
Left subiculum	—	*r* = −0.41, *P* = 0.02*	*r* = 0.16, *P* = 0.96	*r* = −0.42, *P* = 0.10	*P* = 0.36; *d* = −0.55
Left cornu ammonis 1	—	*r* = −0.31, *P* = 0.04*	*r* = −0.04, *P* = 0.96	*r* = −0.32, *P* = 0.16	*P* = 0.46; *d* = −0.38
Left presubiculum	—	*r* = −0.33, *P* = 0.04*	*r* = 0.26, *P* = 0.96	*r* = −0.26, *P* = 0.26	*P* = 0.36; *d* = −0.50
Left molecular layer	—	*r* = −0.30, *P* = 0.04*	*r* = −0.14, *P* = 0.96	*r* = −0.24, *P* = 0.32	*P* = 0.46; *d* = −0.36
Left GC-ML-DG	—	*r* = −0.20, *P* = 0.17	*r* = 0.02, *P* = 0.96	*r* = −0.19, *P* = 0.32	*P* = 0.46; *d* = −0.28
Left cornu ammonis 3	—	*r* = −0.18, *P* = 0.21	*r* = −0.12, *P* = 0.96	*r* = −0.18, *P* = 0.32	*P* = 0.52; *d* = −0.20
Left cornu ammonis 4	—	*r* = −0.21, *P* = 0.17	*r* = −0.02, *P* = 0.96	*r* = −0.18, *P* = 0.32	*P* = 0.46; *d* = −0.28
*Hippocampus subfield volumes and Glx level*					
Left whole hippocampus	*r* = −0.12, *P* = 0.47	*r* = 0.14, *P* = 0.32	*r* = 0.47, *P* = 0.10	*r* = 0.03, *P* = 0.88	—
Left hippocampal tail	*r* = 0.04, *P* = 0.80	*r* = 0.22, *P* = 0.69	*r* = 0.29, *P* = 0.43	*r* = 0.25, *P* = 0.67	—
Left subiculum	*r* = −0.20, P = 0.80	*r* = 0.06, *P* = 0.69	*r* = 0.29, *P* = 0.43	*r* = 0.06, *P* = 0.88	—
Left cornu ammonis 1	*r* = −0.08, *P* = 0.89	*r* = 0.06, *P* = 0.69	*r* = 0.22, *P* = 0.47	*r* = 0.03, *P* = 0.88	—
Left presubiculum	*r* = −0.09, *P* = 0.80	*r* = 0.10, *P* = 0.69	*r* = 0.44, *P* = 0.26	*r* = 0.05, *P* = 0.88	—
Left molecular layer	*r* = −0.22, *P* = 0.80	*r* = 0.13, *P* = 0.69	*r* = 0.51, *P* = 0.26	*r* = 0.04, *P* = 0.88	—
Left GC-ML-DG	*r* = −0.08, *P* = 0.80	*r* = 0.08, *P* = 0.69	*r* = 0.49, *P* = 0.26	*r* = −0.22, *P* = 0.67	—
Left cornu ammonis 3	*r* = −0.06, *P* = 0.80	*r* = 0.07, *P* = 0.69	*r* = 0.26, *P* = 0.43	*r* = −0.03, *P* = 0.88	—
Left cornu ammonis 4	*r* = −0.05, *P* = 0.80	*r* = 0.06, *P* = 0.69	*r* = 0.45, *P* = 0.26	*r* = −0.20, *P* = 0.67	—

Pearson partial correlation coefficients were used to assess relationships between the left hippocampus subfields and DUP or glutamate (with controlling variables, for DUP: age, gender, and ICV; for glutamate level: age, gender, tobacco taking (pack-day), and ICV); all statistical analyses were controlled for multiple comparisons by false detection rate correction.

*FEP* first episode psychosis’s patients, *DUP* duration of untreated psychosis (long > 12 months > short), *BPRS* brief psychiatric rating scale, *GC-ML-DG* granular cell of the dentate gyrus, *d* Cohen’s *d*.

### Relationship between Glx and hippocampal volumes

In both FEP and HC, there were no significant relationship between Glx levels and hippocampal volumes (Table 3).

### Clinical and behavioral relationships

#### Memory function: relationship with DUP, volume, and Glx

There were no significant relationship between the memory subscales of the RBANS and DUP, hippocampal volumes or Glx levels for the HC, the FEP, or the short and long DUP subgroups (Supplementary Tables 2 and 3).

#### Symptoms: relationship with DUP, volume, and Glx

Across the entire FEP sample, after multiple comparison correction, lower severity of positive symptoms were associated with longer DUP (*r* = −0.34, *P* = 0.03), but not severity of negative (*r* = −0.09, *P* = 0.52) or general symptoms (*r* = −0.22, *P* = 0.16). Across the DUP subgroups, no significant correlations were found (Supplementary Tables 3 and 4).

There were no significant associations between positive or negative symptoms, and volume (Supplementary Table 4) or Glx levels for the entire FEP sample or for DUP groups (Supplementary Table 3).

## Discussion

To our knowledge, were are the first to simultaneously evaluate hippocampal glutamate levels and volumes, including subfields, in antipsychotic-naive FEP in order to shed light into the neurobiological underpinnings of the relationship between longer DUP and poorer clinical outcomes. In medication-naive FEP, we found that hippocampal Glx levels were significantly higher in those who underwent a long DUP (>12 months) compared to those with a short DUP, and compared to HC. In addition, we found that smaller hippocampal volumes, including CA1, ML, tail, subiculum, and presubiculum, were significantly associated with longer DUP. However, we found no significant association between hippocampal Glx levels, and hippocampal volume or subfields. Our results strongly suggest that one or several pathophysiological processes underlie the DUP.

### Glutamate levels in left hippocampus

Contrary to our hypothesis, we found no significant differences in levels of Glx between medication-naive FEP and HC in left hippocampus. This is in agreement with another study in a small sample of medication-naive FEP (*n* = 15) where no group difference in Glx levels was identified in the medical temporal cortex^60^. This is in contrast to our prior study, where elevated hippocampus Glx levels was found in a group of unmedicated patients with SZ (refs. ^32–34^). Illness chronicity or prior exposure antipsychotic medication may affect hippocampus Glx. In FEP, Glx levels showed greater variance compared to controls, suggesting heterogeneity in glutamatergic metabolism in FEP. It is possible that only a subset of patients have developed a hyperglutamatergic state^61,62^. Our findings underscore the importance of considering heterogeneity when characterizing psychosis spectrum patients in the early illness stages.

### Association between left hippocampus glutamate level and DUP

Because there is evidence that the association of DUP with outcome is more pronounced for longer DUP (>12 months) compared to shorter DUP (ref. ^4^), we dichotomized our FEP patients based on that cutoff value. We found increased hippocampus Glx levels in those with a DUP > 12 months, compared to those with a DUP < 12 months, and compared to HC. The two DUP subgroups were not significantly different based on demographic characteristics or symptom burden, indicating difference in Glx levels was not related to these variables (Table 1). Our results are in line with the report that higher hippocampus glutamate levels in clinical high-risk individuals for psychosis were associated with a poor functional outcome^35^.

### Left hippocampus volume and subregions in FEP compared to HC

Here, we report significantly lower whole left hippocampus volumes, as well as CA1, CA4, GC-ML-DG, subiculum, and presubiculum in antipsychotic-naive FEP patients compared to HC. While volume deficits of the hippocampus in SZ have been consistently reported [21, 26, 28, 29, 56, 57], only two prior studies measured hippocampal volumes in antipsychotic-naive FEP (refs. ^31,63^). Consistent with our findings, one of them^31^ observed decreased hippocampal volumetric integrity in a cohort of 71 FEP, but they did not measure subfield volumes. The other^63^, reported no significant change in total hippocampal volumes and significantly greater subfield volumes in the ML, granular layer, and CA4 in a cohort of 41 FEP. Interestingly, Ho and colleagues^30^ measured hippocampal volumes and subfields in a large group of patients with SZ in the early stages of illness (mean duration of illness of 7 years), and found deficits in hippocampal whole volume and CA1, but not in other subfields. In addition, they measured hippocampal subfields in an older cohort (mean duration of illness of 18 years) of patients and report deficits in all subfields. It is not clear why this study detected deficits limited to the CA1 region in the early stage psychosis cohort, while we observed more extensive deficits in medication-naive FEP. This cohort was collected in Singapore, while ours was collected from the southeast of the US. It is possible that differences in DUP, stress, or other environmental factors could explain those differences. Another possibility is their hippocampal segmentation was obtained using T1-weighted image, and not T1- and T2-weighted images like we did, which has been shown to improve segmentation^55^. More consistent with our results are the report of deficits in CA1, CA2/3, and CA4/DG in ultra-high-risk subjects compared to HC, deficits that were not as extensive as in a group of SZ patients^21^. However, because ~2/3 of the high-risk population will not convert to psychosis, the interpretation of high-risk studies is complicated. In high-risk individuals who were followed until conversion to psychosis, elevated cerebral blood volume in CA1 region, thought to represent increased metabolism secondary to elevated neuronal activity, predicted subsequent conversion^64^. The progression of atrophy from CA1 to other hippocampal subfields could originate from the particularly vulnerability of CA1 to dysregulation of glutamatergic neurotransmission and excitotoxic injury^30,65,66^.

In summary, our results support that hippocampal abnormalities are already present at psychosis onset but indicate a more extensive reduction in hippocampal subfield volumes in early psychosis than previously thought.

### Relationship between left hippocampus subregions and DUP

We found significant associations between longer DUP and smaller whole hippocampus volumes, as well as CA1, ML, subiculum, presubiculum, and tail. Our findings are consistent with those of Goff et al.^31^ who reported that, in FEP, longer DUP was associated with accelerated hippocampal atrophy over just 8 weeks of antipsychotic treatment, suggesting a persistent effect of DUP on brain structure. These results are consistent with evidence of a damaging effect of DUP on other brain structures^31,67^. Although we did not find an association between hippocampal volumes and memory performances, another study did find associations between hippocampus subregions and cognitive function in high risk and SZ (ref. ^21^). Taken together these data could potentially explain how longer DUP acting through deficits in hippocampus subfields is linked to worse outcome in FEP patients^1,4^.

### Glutamate as a candidate to explain the hippocampus atrophy

We did not report association between whole hippocampus or subfield volumes and Glx levels. Our prior study in unmedicated SZ suggested that alterations in hippocampus glutamatergic neurotransmission may play a role in hippocampus deficits^32^. The lack of association may be the consequence of a nonoptimal overlap between the MRS voxel and the hippocampus volumes. It is also possible that there is a threshold value for a toxic effect of psychosis, rather than a linear relationship between DUP and a neurotoxic effect^36^. Other mechanism, such as increased dopaminergic activity^68^, or persistent catecholaminergic and hypothalamic pituitary adrenal axis activity^69^ have been proposed as causal factors for the association of DUP with poor outcome.

### Limitations

Our findings need to be placed in the context of several strengths and limitations. One of the major strengths here is that all FEP were enrolled at the time of first treatment contact that allowed us to mitigate possible confounds of antipsychotic medication exposure. We also recruited a control group that was carefully matched on key demographic variables, and we controlled for several possible confounding variables (e.g., total ICV) during statistical analyses. We determined DUP with a clinical interview. A quantitative review comparing methods of assessing DUP found that clinical interviews are no less reliable than standardized assessment tools^70^. While our MRS acquisition sequence does have some drawbacks such as J-modulation and T2 relaxation effects on the spectrum, as well as a water signal that is highly T2-weighted and sensitive to cerebrospinal fluid contamination^52^, a significant advantage of these acquisition parameters is that it allows us put findings in context of a number of our previous studies for which we used the same acquisition parameters^32,71,72^. Regarding the hippocampus segmentation, the previous version of computerized segmentation of the hippocampus by Freesurfer was controversial^73^, but the new version, by using high-resolution T2-weighted MR imaging, addresses these criticisms^55^.

### Summary

Here, we demonstrate evidence of lower hippocampal subfield volumes and altered glutamatergic metabolism in those with longer DUP, suggesting possible biological mechanisms underlying the clinical observation that patients with longer DUP suffer from worse overall outcomes. Our data highlight the critical need for reducing the DUP and for early pharmacological intervention, with the hope to prevent structural deficits and improve clinical outcomes.

## Supplementary information

Supplementary data
