# Supplementary material for: Hippocampal glutamate and hippocampus subfield volumes in antipsychotic-naive first episode psychosis subjects and relationships to duration of untreated psychosis
Source: Transl Psychiatry. 2020 May 12;10:137. doi: 10.1038/s41398-020-0812-z (PMC7217844; doi:10.1038/s41398-020-0812-z)
Supplement: Supplementary file 1 — Supplementary data [file 41398_2020_812_MOESM1_ESM.docx]

# Supplementary data

The supplementary information includes MRI scan parameters, MRS supplementary information, supplementary tables and references.

## Data acquisition and preprocessing

Imaging was performed on a 3T whole-body Siemens MAGNETOM Prisma MRI scanner equipped with a 20-channel head coil. A high resolution anatomical T1-weighted magnetization prepared rapid acquisition gradient-echo (MPRAGE) structural scan was acquired for anatomical reference and morphological analyses (TR/TE = 2400/2.22ms; inversion time = 1000ms; flip angle = 8°; GRAPPA factor = 2,208 slices, voxel size = 0.8mm isotropic; and 256 × 256 matrix). A T2-weighted (TR/TE = 3200/563.0ms, flip angle 8°, 256 x 256 matrix, 0.8 mm isotropic voxels) was also acquired for hippocampus subfield segmentation.

### ^1^H-MRS Data Processing

Glx levels were calculated relative to the unsuppressed voxel water and expressed in institutional units (I.U., approximately millimolar) according to the concentration formula of Scheidegger and colleagues ^1^, where in our case the number of protons is 7 for NAA, 6 for Cr, 13 for Cho and 2 for the C4 resonance of Glx, and corrected according to partial volume corrections.

## Supplementary tables

| Supplementary Table 1. Statistical comparisons of right hippocampal subfield volumes (mm^3^) in FEP and HC | | | | | | |
| --- | --- | --- | --- | --- | --- | --- |
|  | HC (n=41) |  | FEP (n=54) | t(85.7) | *P at FDR 0.05* | Cohen’s d |
| Right whole hippocampus | 3551.90 (384.6) |  | 3351.07 (343.9) | 2.66 | <0.01* | 0.55 |
| Right hippocampal tail | 595.81 (78.12) |  | 560.58 (70.55) | 2.28 | 0.05* | 0.48 |
| Right subiculum | 433.99 (52.28) |  | 408.44 (40.63) | 2.61 | 0.03* | 0.55 |
| Right cornu ammonis 1 | 683.31 (88.89) |  | 629.41 (86.10) | 2.988 | 0.01* | 0.62 |
| Right presubiculum | 301.85 (36.36) |  | 277.16 932.16) | 3.47 | <0.01* | 0.72 |
| Right molecular layer | 530.19 (62.44) |  | 509.56 (60.31) | 1.63 | 0.17 | 0.34 |
| Right GC-ML-DG | 314.63 (40.32) |  | 303.64 (35.06) | 1.41 | 0.22 | 0.29 |
| Right cornu ammonis 3 | 228.73 (31.83) |  | 220.64 (32.46) | 1.22 | 0.26 | 0.25 |
| Right cornu ammonis 4 | 259.52 (32.90) |  | 252.32 (28.94) | 1.12 | 0.27 | 0.27 |
|  | FEP subgroup | | | | | |
|  | Long DUP (n=17) |  | Short DUP (n=37) | t(26.42) | *P at FDR 0.05* | Cohen’s d |
| Right whole hippocampus | 3283.84 (360.10) |  | 3381.88 (336.71) | 1.45 | 0.35 | -0.28 |
| Right hippocampal tail | 547.29 (74.87) |  | 566.67 | -0.91 | 0.59 | -0.27 |
| Right subiculum | 396.37 (39.72) |  | 413.99 (40.35) | -1.51 | 0.57 | -0.44 |
| Right cornu ammonis 1 | 615.59 (91.01) |  | 635.75 (84.26) | -0.77 | 0.59 | -0.23 |
| Right presubiculum | 263.59 (32.24) |  | 283.41 (30.56) | -2.13 | 0.32 | -0.63 |
| Right molecular layer | 504.79 (69.81) |  | 511.75 (56.32) | -0.36 | 0.82 | -0.11 |
| Right GC-ML-DG | 297.61 (34.66) |  | 306.42 (35.37) | -0.86 | 0.59 | -0.25 |
| Right cornu ammonis 3 | 219.81 (26.15) |  | 221.02 (35.31) | -0.14 | 0.89 | -0.4 |
| Right cornu ammonis 4 | 247.41 (26.85) |  | 254.58 (29.93) | -0.88 | 0.59 | -0.25 |
| ^HC: Healthy controls; FEP: First episode psychosis's patients; DUP: Duration of untreated psychosis (long>12 months>short); All analyses were controlled for multiple comparisons by false detection rate correction method. GC-ML-DG: Granular cell of the dentate gyrus.^ | | | | | | |

| Supplementary Table 2. Correlations between left hippocampus subfield volumes and RBANS. | | | | | | | |
| --- | --- | --- | --- | --- | --- | --- | --- |
|  | HC |  | FEP all |  | FEP subgroup | | |
|  |  |  |  |  | Long DUP |  | Short DUP |
|  | n=31 |  | n=48 |  | n=15 |  | n=33 |
| *Hippocampus subfield volumes and RBANS (total)* |  |  |  |  |  |  |  |
| Left whole hippocampus | r = -0.04, *P* = 0.83 |  | r = 0.13, *P* = 0.40 |  | r = 0.54, *P* = 0.07 |  | r = 0.02, *P* = 0.90 |
| Left hippocampal tail | r = -0.02, *P* = 0.90 |  | r = -0.07, *P* = 0.71 |  | r = 0.22, *P* = 0.48 |  | r = -0.19, *P* = 0.90 |
| Left subiculum | r = -0.16, *P* = 0.90 |  | r = 0.21, *P* = 0.63 |  | r = 0.46, *P* = 0.17 |  | r = 0.24, *P* = 0.90 |
| Left cornu ammonis 1 | r = -0.09, *P* = 0.90 |  | r = 0.16, *P* = 0.63 |  | r = 0.55, *P* = 0.12 |  | r = 0.08, *P* = 0.90 |
| Left presubiculum | r = 0.06, *P* = 0.90 |  | r = 0.13, *P* = 0.63 |  | r = 0.37, *P* = 0.27 |  | r = 0.13, *P* = 0.90 |
| Left molecular layer | r = -0.07, *P* = 0.90 |  | r = 0.03, *P* = 0.86 |  | r = 0.52, *P* = 0.13 |  | r = -0.11, *P* = 0.90 |
| Left GC-ML-DG | r = 0.04, *P* = 0.90 |  | r = 0.13, *P* = 0.63 |  | r = 0.58, *P* = 0.12 |  | r = -0.04, *P* = 0.90 |
| Left cornu ammonis 3 | r = 0.13, *P* = 0.90 |  | r = 0.08, *P* = 0.71 |  | r = 0.56, *P* = 0.12 |  | r = -0.07, *P* = 0.90 |
| Left cornu ammonis 4 | r = 0.03, *P* = 0.90 |  | r = 0.14, *P* = 0.63 |  | r = 0.61, *P* = 0.12 |  | r = -0.02, *P* = 0.90 |
| *Hippocampus subfield volumes and RBANS (IM)* |  |  |  |  |  |  |  |
| Left whole hippocampus | r = 0.08, *P* = 0.67 |  | r = 0.06, *P* = 0.68 |  | r = 0.60, *P* = 0.04* |  | r = -0.11, *P* = 0.60 |
| Left hippocampal tail | r = -0.05, *P* = 0.79 |  | r = -0.11, *P* = 0.91 |  | r = 0.33, *P* = 0.30 |  | r = -0.28, *P* = 0.92 |
| Left subiculum | r = -0.07, *P* = 0.79 |  | r = 0.18, *P* = 0.91 |  | r = 0.52, *P* = 0.13 |  | r = 0.13, *P* = 0.92 |
| Left cornu ammonis 1 | r = 0.09, *P* = 0.79 |  | r = 0.14, *P* = 0.91 |  | r = 0.69, *P* = 0.07 |  | r = -0.01, *P* = 0.97 |
| Left presubiculum | r = 0.14, *P* = 0.79 |  | r = 0.08, *P* = 0.91 |  | r = 0.35, *P* = 0.30 |  | r = 0.01, *P* = 0.97 |
| Left molecular layer | r = 0.12, *P* = 0.79 |  | r = -0.01, *P* = 0.97 |  | r = 0.67, *P* = 0.07 |  | r = -0.19, *P* = 0.92 |
| Left GC-ML-DG | r = 0.10, *P* = 0.79 |  | r = 0.02, *P* = 0.97 |  | r = 0.46, *P* = 0.17 |  | r = -0.13, *P* = 0.92 |
| Left cornu ammonis 3 | r = 0.24, *P* = 0.79 |  | r = 0.06, *P* = 0.91 |  | r = 0.60, *P* = 0.10 |  | r = -0.07, *P* = 0.92 |
| Left cornu ammonis 4 | r = 0.10, *P* = 0.79 |  | r = 0.07, *P* = 0.91 |  | r = 0.56, *P* = 0.12 |  | r = -0.08, *P* = 0.92 |
| *Hippocampus subfield volumes and RBANS (DM)* |  |  |  |  |  |  |  |
| Left whole hippocampus | r = 0.03, *P* = 0.88 |  | r = 0.06, *P* = 0.68 |  | r = 0.53, *P* = 0.07 |  | r = -0.04, *P* = 0.81 |
| Left hippocampal tail | r = 0.03, *P* = 0.98 |  | r = 0.11, *P* = 0.98 |  | r = 0.31, *P* = 0.38 |  | r = 0.07, *P* = 0.82 |
| Left subiculum | r = -0.05, *P* = 0.98 |  | r = 0.18, *P* = 0.98 |  | r = 0.71, *P* = 0.07 |  | r = 0.12, *P* = 0.75 |
| Left cornu ammonis 1 | r = 0.01, *P* = 0.98 |  | r = 0.01, *P* = 0.98 |  | r = 0.28, *P* = 0.38 |  | r = -0.01, *P* = 0.98 |
| Left presubiculum | r = 0.06, *P* = 0.98 |  | r = 0.02, *P* = 0.98 |  | r = 0.53, *P* = 0.15 |  | r = -0.11, *P* = 0.76 |
| Left molecular layer | r = -0.10, *P* = 0.98 |  | r = -0.05, *P* = 0.98 |  | r = 0.35, *P* = 0.36 |  | r = -0.13, *P* = 0.76 |
| Left GC-ML-DG | r = 0.15, *P* = 0.98 |  | r = 0.01, *P* = 0.98 |  | r = 0.65, *P* = 0.08 |  | r = -0.17, *P* = 0.76 |
| Left cornu ammonis 3 | r = 0.11, *P* = 0.98 |  | r = -0.07, *P* = 0.98 |  | r = 0.37, *P* = 0.36 |  | r = -0.14, *P* = 0.76 |
| Left cornu ammonis 4 | r = 0.14, *P* = 0.98 |  | r = -0.01, *P* = 0.98 |  | r = 0.62, *P* = 0.08 |  | r = -0.13, *P* = 0.76 |
| ^FEP: First episode psychosis's patients; DUP: Duration of untreated psychosis (long>12 months>short); RBANS: Repeatable battery for the assessment of neuropsychological status (IM: Immediate memory, DM: Delayed memory); Pearson partial correlation coefficients were used to assess relationships between the left hippocampus subfields and RBANS (with controlling variables: age, gender and ICV); All statistical analyses were controlled for multiple comparisons by false detection rate correction method. GC-ML-DG: Granular cell of the dentate gyrus.^ | | | | | | | |

| Supplementary Table 3. Correlations between Glx level, DUP and clinical assessment in left hippocampus | | | | | |  |
| --- | --- | --- | --- | --- | --- | --- |
|  | FEP all |  | FEP subgroup | | | |
|  |  |  | Long DUP |  | Short DUP |  |
|  |  | n |  | n |  | n |
| BPRS vs DUP |  |  |  |  |  |  |
| Positive | r = -0.34, *P* = 0.03* | 54 | r = -0.06, *P* = 0.81 | 17 | r = -0.34, *P* = 0.11 | 37 |
| Negative | r = -0.09, *P* = 0.52 | 54 | r = -0.35, *P* = 0.50 | 17 | r = 0.29, *P* = 0.44 | 37 |
| Total | r = -0.22, *P* = 0.16 | 54 | r = -0.15, *P* = 0.81 | 17 | r = -0.11, *P* = 0.49 | 37 |
| BPRS vs [Glx] |  |  |  |  |  |  |
| Positive | r = -0.12, *P* = 0.37 | 54 | r = -0.27, *P* = 0.34 | 17 | r = 0.02, *P* = 0.88 | 37 |
| Negative | r = -0.14, *P* = 0.37 | 54 | r = -0.42, *P* = 0.12 | 17 | r = -0.03, *P* = 0.88 | 37 |
| Total | r = -0.19, *P* = 0.37 | 54 | r = -0.29, *P* = 0.30 | 17 | r = -0.04, *P* = 0.88 | 37 |
| RBANS vs DUP |  |  |  |  |  |  |
| Total | r = 0.10, *P* = 0.81 | 48 | r = -0.28, *P* = 0.47 | 15 | r = -0.13, *P* = 0.72 | 33 |
| Immediate memory | r = -0.01 , *P* = 0.81 | 48 | r = -0.31, *P* = 0.47 | 15 | r = -0.02 , *P* = 0.91 | 33 |
| Delayed memory | r = 0.11, *P* = 0.81 | 48 | r = -0.15, *P* = 0.58 | 15 | r = -0.16, *P* = 0.72 | 33 |
| RBANS vs [Glx] |  |  |  |  |  |  |
| Total | r = 0.26, *P* = 0.13 | 48 | r = 0.07, *P* = 0.81 | 15 | r = 0.22, *P* = 0.38 | 33 |
| Immediate memory | r = 0.08, *P* = 0.61 | 48 | r = -0.07, *P* = 0.81 | 15 | r = 0.10, *P* = 0.59 | 33 |
| Delayed memory | r = 0.26, *P* = 0.13 | 48 | r = 0.16, *P* = 0.81 | 15 | r = 0.21, *P* = 0.38 | 33 |
| ^FEP: First Episode Psychosis's patients; DUP: Duration of untreated psychosis (long>12 months>short); BPRS: Brief Psychiatric Rating Scale; [Glx] = Glutamate concentration; RBANS: Repeatable Battery for the Assessment of Neuropsychological Status; For see the association between BPRS and DUP, Pearson correlation coefficients were used. Pearson partial correlation coefficients were used to assess Glx level with scales (with controlling variables: age, gender, tobacco taking (pack-day) and ICV); All statistical analyses were controlled for multiple comparisons by false detection rate correction method.^ | | | | | | |

| Supplementary Table 4. Correlations between left hippocampus subfield volumes and BPRS. | | | |  |  |
| --- | --- | --- | --- | --- | --- |
|  | FEP all |  | FEP subgroup | | |
|  |  |  | Long DUP |  | Short DUP |
|  | n=54 |  | n=17 |  | n=37 |
| *Hippocampus subfield volumes and BPRS positive* |  |  |  |  |  |
| Left whole hippocampus | r = 0.09, *P* = 0.48 |  | r = 0.32, *P* = 0.27 |  | r = -0.06, *P* = 0.73 |
| Left hippocampal tail | r = 0.18, *P* = 0.92 |  | r = 0.15, *P* = 0.66 |  | r = 0.18, *P* = 0.80 |
| Left subiculum | r = 0.17, *P* = 0.92 |  | r = 0.58, *P* = 0.25 |  | r = -0.03, *P* = 0.91 |
| Left cornu ammonis 1 | r = 0.02, *P* = 0.98 |  | r = 0.16, *P* = 0.66 |  | r = -0.12, *P* = 0.91 |
| Left presubiculum | r = 0.12, *P* = 0.92 |  | r = 0.37, *P* = 0.48 |  | r = 0.02, *P* = 0.91 |
| Left molecular layer | r = -0.01, *P* = 0.98 |  | r = 0.13, *P* = 0.66 |  | r = -0.12, *P* = 0.91 |
| Left GC-ML-DG | r = 0.11, *P* = 0.92 |  | r = 0.36, *P* = 0.47 |  | r = -0.08, *P* = 0.91 |
| Left cornu ammonis 3 | r = -0.01, *P* = 0.98 |  | r = 0.17, *P* = 0.66 |  | r = -0.18, *P* = 0.91 |
| Left cornu ammonis 4 | r = 0.07, *P* = 0.98 |  | r = 0.34, *P* = 0.47 |  | r = -0.12, *P* = 0.91 |
| *Hippocampus subfield volumes and BPRS negative* | |  |  |  |  |
| Left whole hippocampus | r = -0.04, *P* = 0.76 |  | r = -0.18, *P* = 0.53 |  | r = -0.12, *P* = 0.51 |
| Left hippocampal tail | r = -0.01, *P* = 0.96 |  | r = 0.04, *P* = 0.94 |  | r = -0.16, *P* = 0.92 |
| Left subiculum | r = 0.02, *P* = 0.96 |  | r = -0.12, *P* = 0.94 |  | r = -0.01, *P* = 0.93 |
| Left cornu ammonis 1 | r = -0.10, *P* = 0.96 |  | r = -0.40, *P* = 0.82 |  | r = -0.12, *P* = 0.92 |
| Left presubiculum | r = -0.03, *P* = 0.96 |  | r = -0.16, *P* = 0.94 |  | r = -0.07, *P* = 0.92 |
| Left molecular layer | r = -0.03, *P* = 0.96 |  | r = -0.36, *P* = 0.82 |  | r = -0.01, *P* = 0.92 |
| Left GC-ML-DG | r = -0.02, *P* = 0.96 |  | r = -0.02, *P* = 0.94 |  | r = -0.12, *P* = 0.92 |
| Left cornu ammonis 3 | r = 0.01, *P* = 0.96 |  | r = 0.10, *P* = 0.94 |  | r = -0.10, *P* = 0.92 |
| Left cornu ammonis 4 | r = -0.01, *P* = 0.96 |  | r = -0.03, *P* = 0.94 |  | r = -0.08, *P* = 0.92 |
| *Hippocampus subfield volumes and BPRS total* |  |  |  |  |  |
| Left whole hippocampus | r = -0.04, *P* = 0.80 |  | r = 0.03, *P* = 0.92 |  | r = -0.12, *P* = 0.49 |
| Left hippocampal tail | r = 0.14, *P* = 0.80 |  | r = -0.03, *P* = 0.96 |  | r = 0.22, *P* = 0.51 |
| Left subiculum | r = 0.04, *P* = 0.80 |  | r = 0.30, *P* = 0.96 |  | r = -0.10, *P* = 0.70 |
| Left cornu ammonis 1 | r = -0.10, *P* = 0.80 |  | r = -0.09, *P* = 0.96 |  | r = -0.19, *P* = 0.51 |
| Left presubiculum | r = -0.04, *P* = 0.80 |  | r = 0.01, *P* = 0.96 |  | r = -0.07, *P* = 0.69 |
| Left molecular layer | r = -0.06, *P* = 0.80 |  | r = -0.02, *P* = 0.96 |  | r = -0.09, *P* = 0.69 |
| Left GC-ML-DG | r = -0.05, *P* = 0.80 |  | r = 0.14, *P* = 0.96 |  | r = -0.20, *P* = 0.51 |
| Left cornu ammonis 3 | r = -0.08, *P* = 0.80 |  | r = 0.08, *P* = 0.96 |  | r = -0.23, *P* = 0.51 |
| Left cornu ammonis 4 | r = -0.04, *P* = 0.80 |  | r = 0.14, *P* = 0.96 |  | r = -0.18, *P* = 0.51 |
| ^FEP: First episode psychosis's patients; DUP: Duration of untreated psychosis (long>12 months>short); RBANS: Repeatable battery for the assessment of neuropsychological status (IM: Immediate memory, DM: Delayed memory); Pearson partial correlation coefficients were used to assess relationships between the left hippocampus subregions and RBANS (with controlling variables: age, gender and ICV); All statistical analyses were controlled for multiple comparisons by false detection rate correction method. GC-ML-DG: Granular cell of the dentate gyrus.^ | | | | | |

# References

1 Scheidegger O, Wingeier K, Stefan D, Graveron-Demilly D, van Ormondt D, Wiest R *et al.* Optimized quantitative magnetic resonance spectroscopy for clinical routine. *Magn Reson Med* 2013; **70**: 25–32.
